# National Food Surveys: diet and more

**DOI:** 10.11606/s1518-8787.2021055supl1ed

**Published:** 2021-11-22

**Affiliations:** I Universidade Federal de São Paulo Departamento de Políticas Públicas e Saúde Coletiva São Paulo SP Brasil Universidade Federal de São Paulo. Departamento de Políticas Públicas e Saúde Coletiva. São Paulo, SP, Brasil; II Faculdade de Ciências Médicas Santa Casa de São Paulo Departamento de Saúde Coletiva São Paulo sp Brasil Faculdade de Ciências Médicas Santa Casa de São Paulo. Departamento de Saúde Coletiva. São Paulo,SP, Brasil

The study of the social determination of health or diseases along life shows how different aspects of daily routine contribute to various outcomes, from the adequate growth and development in childhood to the early mortality. Among many determinants, we emphasize food consumption in different stages of life^1^.

Encouraging a good diet for good health is one of the most efficient ways to protect people from illnesses, while also helping the environment and public management^2^. Simultaneously, the excessive consumption of ultra-processed foods follows the predominant economic logic of wealth accumulation (for the few) and causes environmental and social degradation^3,4^.

Despite scientific evidence on why a good diet is important to stay healthy and avoid various diseases, study of the relationships between food and nutrients and health continues to face theoretical, methodological and instrumental challenges, as pointed by one of the articles published here.

Therefore, population surveys such as the *Pesquisa de Orçamentos Familiares* (POF – Consumer Expenditure Survey) are essential to monitor food consumption and expenses, dietary patterns, outside meals, and energy and nutrient intake. POF is already in its sixth edition (2017–2018) and has contributed to diverse fields of knowledge in Brazil, including Health.

The 2017–2018 POF brought data from the second *Inquérito Nacional de Alimentação* (INA – National Food Survey) using 24h recalls (R24h), with more than 46.000 individuals from all over the country, and on two non-consecutive days. R24h represents an important improvement for data collection when compared to the first edition of the INA in 2008–2009, which used the two-day food registry from about 30.000 individuals. Two other positive outcomes of the 2017–2018 POF were the expansion of biomarkers to validate consumption data, including research for urinary nitrogen (protein), sodium, and potassium, and the analyses with doubly labeled water to assess energy intake, present in both studies^5^.

Despite advances, the reduction of measurement errors in food surveys, resulting from the many stages (collection, transformation of reported amounts into weight, conversion of food into energy and nutrients), still poses a challenge for studies on food consumption. This supplement includes the first studies that compare data of the two INAs and address some of the most relevant methodological aspects in such studies, also focusing on vital information about diet in Brazil.

Data from 2008–2009 and 2017–2018 shows that, despite methodological differences, Brazilian diet decreased in quality, especially because rice, dairy, beans, meat, and fish are less consumed; meanwhile, ultra-processed foods, which could cause chronic diseases, are still frequently consumed, except for some items such as soft drinks. The latter study shows an increased number of foods with greater diet diversity, which may encourage eating protective foods such as fruits and vegetables.

The supplement also includes articles that compare the two National Food Surveys – part of the Consumer Expenditure Survey, with a 10-year gap – and are helpful to create policies and programs that prevent chronic diseases, encourage healthy eating as essential for good health, food surveillance and security, and monitor unhealthy behaviors. Additionally, they share knowledge about food “preferences”, shaped by the constraints that modern life and economic and financial difficulties represent for many Brazilians, affecting health and the number of chronic diseases in adults and older adults.
