# Supplementary material for: National Food Surveys: diet and more
Source: Rev Saude Publica. 2021 Nov 22;55 Supl 1:1s. doi: 10.11606/s1518-8787.2021055supl1ed (PMC8969488; doi:10.11606/s1518-8787.2021055supl1ed)
Supplement: Translation

# Translation: Inquéritos Nacionais de Alimentação: consumo alimentar e muito mais

**Affiliations:** I São Paulo SP Brasil Universidade Federal de São Paulo. Departamento de Políticas Públicas e Saúde Coletiva. São Paulo, SP, Brasil; II São Paulo SP Brasil Faculdade de Ciências Médicas Santa Casa de São Paulo. Departamento de Saúde Coletiva. São Paulo SP, Brasil

O estudo da determinação social do processo saúde-doença que se desenrola ao longo do ciclo da vida revela como diferentes aspectos do cotidiano contribuem para os mais variados desfechos, desde o adequado crescimento e desenvolvimento na infância até a mortalidade precoce por diferentes males. Dentre os inúmeros determinantes destacam-se a alimentação e a dieta nas diferentes etapas da vid^1^.

A promoção da saúde por meio da boa alimentação é uma das estratégias mais eficientes para proteger as pessoas dos riscos de adoecimento, com efeitos positivos também para o ambiente e para a gestão pública^2^. Contemporaneamente, o consumo excessivo de produtos alimentares ultraprocessados obedece à lógica econômica predominante de acúmulo de riqueza (para poucos) e traz ainda degradação ambiental e social^3,4^.

Apesar das evidências científicas sobre a importância da alimentação saudável não apenas para a manutenção da saúde, mas também para a prevenção de diversas doenças, o estudo das relações entre alimentos, nutrientes e estados de saúde continua apresentando uma série de desafios teóricos, metodológicos e instrumentais, como bem assinalado em um dos artigos ora publicados.

Assim, inquéritos populacionais como a Pesquisa de Orçamentos Familiares (POF) são fundamentais para acompanhar a estrutura de consumo, o gasto com alimentação, os padrões alimentares, o consumo fora do domicílio e a ingestão energética e de nutrientes. Os dados da POF, já em sua sexta edição (2017–2018), têm sido relevantes para as mais diversas áreas de conhecimento no Brasil, dentre as quais a grande área da saúde.

A POF 2017–2018 trouxe os dados do segundo Inquérito Nacional de Alimentação (INA), nesta edição realizado por meio de recordatórios de 24h (R24h), com mais de 46 mil indivíduos em todo o país, e em dois dias não consecutivos. A adoção do R24h representa um importante aprimoramento para a coleta de dados, relativamente à primeira edição do INA em 2008–2009, quando se empregou o registro alimentar de dois dias, realizado por aproximadamente 30 mil indivíduos. Outra boa novidade da edição de 2017–2018 foi a ampliação dos biomarcadores para validação dos dados de consumo, com a inclusão da pesquisa para nitrogênio (proteína) e sódio e potássio urinários, além das análises com água duplamente marcada para avaliar o relato da ingestão de energia, que ocorreu em ambos os estudos^5^.

Apesar dos avanços, a diminuição dos erros de medida em inquéritos alimentares, decorrentes das diversas etapas (coleta, transformação das quantidades relatadas em peso, conversão de alimentos em energia e nutrientes), segue sendo um desafio para estudos de consumo alimentar. Temos neste suplemento os primeiros estudos que puderam contar com a comparação entre dados dos dois INAs e endereçar alguns dos mais relevantes aspectos metodológicos em estudos desse porte, bem como trazer à luz as informações que a sociedade precisa sobre o cenário da alimentação no Brasil.

A comparação dos dados entre os anos de 2008–2009 e 2017–2018, apesar das diferenças metodológicas, revela que houve piora da qualidade da alimentação do brasileiro, notadamente pela diminuição do consumo de arroz, laticínios, feijão, carne e peixe; e, alimentos ultraprocessados, marcadores de risco para doenças crônicas, seguem entre os alimentos mais consumidos, apesar da queda observada para alguns itens, como os refrigerantes. A ampliação do número de alimentos entre os dois estudos revela maior diversidade de itens alimentares, o que pode favorecer estratégias de incentivo ao consumo de alimentos protetores, como as frutas, os legumes e as verduras.

Os diferentes aspectos tratados no conjunto de artigos derivados das comparações entre os dois Inquéritos Nacionais de Alimentação – parte das Pesquisas de Orçamentos Familiares realizadas com intervalo de 10 anos – apresentados neste suplemento, oferecem um amplo painel de informações para a formulação de políticas e programas de prevenção de doenças crônicas, promoção da alimentação saudável como componente essencial para a manutenção da saúde, vigilância e segurança alimentar, monitoramento de comportamentos não saudáveis. Adicionalmente, propiciam o conhecimento sobre as “preferências” alimentares, moldadas pelos constrangimentos que a vida moderna e as dificuldades econômico-financeiras representam para amplas parcelas da população brasileira, com consequências para o estado de saúde e para a carga de doenças crônicas nas populações adultas e idosas.
